# Hijacking the Host Immune Cells by Dengue Virus: Molecular Interplay of Receptors and Dengue Virus Envelope

**DOI:** 10.3390/microorganisms7090323

**Published:** 2019-09-06

**Authors:** Feroza Begum, Sandeepan Das, Debica Mukherjee, Upasana Ray

**Affiliations:** 1 CSIR-Indian Institute of Chemical Biology; 4, Raja S.C. Mullick Road, Jadavpur, Kolkata 700032, West Bengal, India; 2 Academy of Scientific and Innovative Research (AcSIR), Ghaziabad 201002, India

**Keywords:** DENV, tropism, receptors, entry, immune cells

## Abstract

Dengue virus (DENV) is one of the lethal pathogens in the hot climatic regions of the world and has been extensively studied to decipher its mechanism of pathogenesis and the missing links of its life cycle. With respect to the entry of DENV, multiple receptors have been recognized in different cells of the human body. However, scientists still argue whether these identified receptors are the exclusive entry mediators for the virus. Adding to the complexity, DENV has been reported to be infecting multiple organ types in its human host. Also, more than one receptor in a particular cell has been discerned to take part in mediating the ingress of DENV. In this review, we aim to discuss the different cells of the human immune system that support DENV infection and their corresponding receptors that DENV deploy to gain access to the cells.

## 1. Introduction

The genus *Flavivirus* includes enveloped viruses (approximately 50 nm in diameter) containing a positive sense, single-stranded RNA (approximately 11 kb in size) genome. Dengue virus (DENV) is one such arbovirus having a genome encoding three structural proteins (C, prM/M, E) and seven non-structural proteins (NS1, NS2A, NS2B, NS3, NS4A, NS4B and NS5) [1]. The envelope of the mature virus contains 180 copies of two glycoproteins, prM and E [2]. Depending on the heterogeneity in these two surface proteins, DENV is broadly classified into four serotypes and each serotype is further distinguished into different genotypes [3].

DENV, being an arbovirus, entirely depends on its insect vectors *Aedes aegypti* and *Aedes albopictus* for circulation in the environment and ultimately reaches its human host for extensive proliferation.

Once DENV gains access to the host, it infects different organs and replicates in multiple cells. DENV exploits various cellular receptors to enter the cells. Although various cellular receptors have been identified as receptors for virus entry, none of them have been recognized as a universal receptor for DENV entry. Here, we will discuss the immune cells that are known to harbor DENV during the disease progression and the corresponding receptors studied so far. It remains an underexplored field and we are yet to nail down the primary receptor/s involved in the entry process. A better understanding of the receptor usage might further help designing specific antiviral candidate/s against DENV infection.

## 2. DENV Entry Receptors in Cells of the Immune System

### 2.1. Dendritic Cells (DCs)

Broadly, there are two subsets of DCs found in the mammalian system: Interferon (IFN) secreting, blood and lymphoid tissue-resident plasmacytoid DC (pDC) and antigen-presenting, lymphoid and non-lymphoid tissue-resident myeloid or conventional dendritic cells (mDCs or cDCs). The antigen-presenting property of DC has been exploited by DENV to disseminate from the skin to various lymphoid organs. Also, a common monocyte-DC precursor differentiates to give rise to tissue-resident macrophages and monocyte-derived DCs (moDC) which are non-conventional DCs [1].

The immature DCs (iDCs) particularly in the skin Langerhans cells (LCs), dermal cDC and moDC} and in blood have been shown to be more susceptible to DENV infection than mature DC, and DENV infects these cells independent of Fcγ receptor [4,5,6]. pDCs are not found to be DENV targets *in vitro* as significantly lower levels of DENV replication was observed when compared to moDC [7,8]. Previous experiments proved LCs in the epidermis to be the primary targets of DENV in the skin, however, subsequent experiments suggested that DENV is probably released in the dermal layer of the skin affecting its resident cells first [4,9,10]. Hence, the route by which epidermal-resident cells (LC and keratinocytes) get infected is still unclear. Studies done by Duangkhae 2018 showed that DENV likely mediates LC migration to the dermis where these cells further get infected [11]. Also, studies done by other groups indicate dermal cDCs and macrophages to play a more significant role than LCs in DENV spread [10,12]. 

The most extensively studied DC receptors are DC-SIGN(CD209) [4,13,14,15], Mannose receptor (MR) [16,17], Langerins [18,19] and Fcγ receptors [7,20,21]. Other potential receptors expressed in DC include TIM3, TIM4 [22,23,24] and AXL [25]. 

DC-SIGN, a C type lectin pathogen recognition receptor, is highly expressed in immature DCs like resident dermal DCs (CD14+), monocyte-derived DC in the dermis, DC in the lymph node, thymus and lungs, myeloid DCs in blood and also in dermal and alveolar macrophages [7,8,10,13,15,26,27]. Although, in presence of Ca^2+^ the ‘carbohydrate recognition domain’ (CRD) of DC-SIGN has been shown to interact with the high mannose oligosaccharides present in Asn67 residue of DENV E, DC-SIGN is also reported to bind to the other branched glycans containing terminal fucose residues [28,29,30,31,32]. 

The importance of DC-SIGN as a DENV entry receptor was highlighted when its expression in various cells lines rendered these cells permissive to DENV infection [13,15,28]. The mechanism by which DC-SIGN mediates DENV entry was further studied by Liu et al. 2017. By using live-cell imaging on DENV infected MX-DC-SIGN cells, the researchers showed that DC-SIGN and DENV, after forming a complex, migrate towards clathrin-coated pits and get endocytosed. However, the mutants lacking the internalization domain (DC-SIGN-3A) or the one containing a partial cytoplasmic domain (DC-SIGN-Δ35) when expressed in MX-DC-SIGN cells still favored DENV infection, although to a lesser extent than the intact DC-SIGN. Other groups also found similar results when the mutant DC-SIGN (without cytoplasmic tail) in HEK-293T cells could still enhance DENV infection [28,33]. Hence, the role of DC-SIGN in mediating the DENV infection has been interjected to be an attachment factor for DENV, which concentrates the virus on the cell surface and presents it to a mysterious receptor, further ushering DENV to the endosomal compartment [13].

Studies done by Cerny et al. 2014 on single-cell suspensions of healthy human skin discovered LCs, dermal macrophages and CD14+DCs to be highly infected as compared to the other subsets of dermal cDCs (CD1c+ and CD141+DCs) [9]. On further study, they showed that CD14+DC being highly susceptible to DENV infection, expressed both DC-SIGN (CD209) and mannose receptor (MR) (CD206). However, CD1c+DC showing lesser susceptibility to DENV expressed only mannose receptor (CD206) and CD141+DC being least susceptible expressed none of these receptors. Dermal macrophages also express both DC-SIGN (CD209) and mannose receptor (CD206) on their cell surface and hence are highly permissive to DENV [27]. Hence, both receptors (DC-SIGN and MR) seem to work together to mediate DENV entry in these cells. MR is constitutively internalized via an endocytic or phagocytic pathway and hence may act as a DENV entry receptor for DC and macrophages, whereas DC-SIGN is mostly confined to the cell surface and work as an important attachment factor [17].

Langerin (CD207) is another C type lectin receptor similar to DC-SIGN and is predominantly expressed in Langerhans cells in the epidermis [18,19]. It also specifically recognizes mannose and fucose glycans along with GlcNAc moieties on the DENV E protein [34]. DENV uses this receptor to gain access to LCs in the skin where it proliferates for further dissemination [4].

TIM3 and TIM4 are another group of receptors that are expressed on the surface of DC and might facilitate DENV entry in these cells [22,23]. TIM3 and TIM4 have been observed to play an important role in phagocytosis of apoptotic cells and in particular, TIM-4 was detected in immature DCs and macrophages of the spleen [24]. These receptors have been studied to mediate DENV entry in transfected cell lines where TIM3 has been perceived to play a less significant role than TIM1 and TIM4 in mediating the entry process [35]. TAM receptors (TYRO3, AXL and MER) particularly AXL, also involved in the uptake of the apoptotic cells, has been observed to be expressed on the surface of Langerhans cells early during its differentiation and might play a significant role in mediating DENV infection in these cells [25]. 

Antibody-dependent enhancement (ADE) is the mechanism by which the heterologous antibodies (IgG), irrespective of the neutralizing capabilities, surround DENV during the secondary infection and present it to FcγR bearing cells to enhance DENV infection [8,36,37,38,39,40,41,42,43].

In the case of ADE, mature DC and macrophages showed enhanced infectivity at a low concentration of heterologous antibodies. Hence, immature DCs (iDC) and mature DCs (maDC) were observed to play a distinct role in the primary and secondary infection, and cell tropism was found to be slightly different in two conditions. In primary infection, iDC were infected most followed by maDC and macrophage, whereas in secondary infection, in presence of heterologous anti DENV antibodies the macrophage is infected most followed by maDC and iDC [44].

FcγRIIa and FcγRIIb are the two receptors that are expressed on the hematopoietic cells and interact with the opsonizing antibodies (IgG) surrounding DENV particle for its enhanced phagocytosis. The LCs, immature moDCs and dermal DCs, as they express high levels of langerins (LC) and DC-SIGN (moDCs and dermal DCs) respectively, become primary targets of DENV in absence of enhancing antibodies [4,5,15]. Despite this, they do not play any role in ADE even though they express FcγRIIA highly as elevated levels of expressed DC-SIGN plays a dominant role and mediate DENV entry in these cells [7,20]. In contrast to this, mature moDCs gets infected by DENV moderately during primary infection as they express the lesser amount of DC-SIGN but inflated levels of FcγRIIA on their surface, showing significantly high capacity for ADE [7,20]. 

### 2.2. Monocytes and Macrophages 

Monocytes and macrophages are the primary targets of DENV along with the dendritic cells. It has been reported that macrophages in lymphoid and non-lymphoid tissues are the major targets of DENV replication during the later period of infection. They are also the primary reservoirs of DENV after its dissemination from the skin. DENV was found to replicate in macrophages of different organs namely, Kupfer cells in the liver, alveolar macrophages in the lungs, macrophages of lymphoid organs (spleen, lymph node and thymus) dermal macrophages, microglial cells and monocytes in peripheral blood [9,37,45,46,47,48].

Experiments in mice indicate that after DENV infects the skin, the inflammatory Ly6C+ monocytes were recruited to the skin-replenishing LCs in the epidermis and Ly6C+ CD11b+ moDC in the dermis which were efficiently targeted by DENV [7,10]. These studies indicate that this observation possibly holds true in case of humans, where blood-derived monocytes migrate to the site of infection in the skin and act as another reservoir of DENV for replication [7,9,10,49]. 

Cell surface receptors that help in DENV tropism in monocytes and macrophage include mannose receptor (CD205) [16,17,50], CD14-associated protein [51,52], HSP70/HSP90 [53,54], DC-SIGN(CD209) [7,10,15,50] and CD300a [55,56], AXL, TIM4 [22,24,57] and PD1[58].

Mannose receptor (MR) is another C type lectin, found in both macrophages and DC and has a multi-domain structure [16,17,50]. MR binds specifically to the carbohydrate moieties terminating in mannose, fucose and N-acetyl Glucosamine (NAG) residues as found in Asn67 of DENV E glycoproteins [16,17,50]. Mannose receptor has been shown by Miller et al. 2008 to be an important receptor for DENV entry in human macrophages. Pre-treatment of monocytes with type 2 cytokines enhanced the surface expression of MR and DC-SIGN on human monocyte-derived macrophages which led to the increased percentage of infected cells. DC-SIGN, which is known to play a role in DENV attachment to DC, also has some role to play in macrophage susceptibility to DENV, probably acting as an additional attachment factor for these cells [17]. However, dermal and alveolar macrophages are the only macrophages that possess cell surface DC-SIGN and hence may mediate DENV infection in these cells in cooperation with MR [26,27].

CD14 is a cell surface glycoprotein, expressed predominantly on the surface of monocytes and macrophages and possesses a high affinity for LPS [59]. It remains associated with the low-affinity transmembrane proteins that show signal-transducing properties [52,59]. *In vitro* infection model studies on monocytes and macrophages have shown a role of CD14 or its associated molecules in DENV mediated infection, as pre-treatment of these cells with LPS before DENV infection suppressed the infection markedly [51,52]. The decrease in infection was inspected not due to LPS mediated release of cytokines but due to blockage of CD14 and its associated cell surface molecules by LPS [52]. However, the outcome of LPS pre-treatment before DENV infection was perceived to show both strain and cell-specific effect on DENV infection [51].

Cell surface proteins like HSP70 and HSP90 are known to be a part of the receptor complex helping in DENV tropism in human monocytes and neurons [53,54]. Hsp90 and Hsp70 (74/84 kDa molecule) isolated from neuroblastoma cell line SK-SY-5Y, U937 cells and human peripheral monocytes/macrophages, was observed to interact with DENV-2 strain 16681 E protein and pre-treatment with anti Hsp 70/anti Hsp90 antibodies reduced DENV infection in these cells [54].

It has been observed that phospholipid receptors like TIM, TAM and CD300a expressed on the surface of phagocytes recognize phospholipids like phosphatidylethanolamine (PE) and phosphatidylserine (PS) expressed on the surface of apoptotic bodies and mediate their phagocytosis [24,25,35,56,57]. This mechanism has been exploited by DENV to interact with such receptors as the DENV membranes express such phospholipids which it acquires during the process of virus budding from ER [35,55,60]).

The T cell/transmembrane, immunoglobulin and mucin (TIM) gene family include three members in humans (TIM-1, TIM-3 and TIM-4) and these receptors are expressed in different cells with slightly different functions.TIM1 is highly expressed on T-helper 2 (Th2) cells and are important for T cell activation, TIM 3 is highly expressed on Th1, Tc1 cells and DC mediating phagocytosis of the apoptotic cells and cross-presentation of antigen and TIM4 is expressed in antigen-presenting cells (APC) and has a role in phagocytosis of apoptotic cells and immune tolerance [22,23]

TIM 1, TIM 4 and to a lesser extent TIM3 were found to enhance mosquito-derived DENV2-JAM infection when expressed in 3T3 and Vero cell lines and a direct interaction between TIM receptors and DENV virions was observed in a Ca^2+^ dependent manner [35]. Furthermore, the authors showed that TIM receptors (TIM 1 and TIM 4) expressing 293T cells recognized PS expressed on DENV virion envelope to mediate its entry in the cells. The role of TIM1 in DENV infection was inferred by Meertens et al. 2012 and Dejarnac et al. 2018 where the significance of TIM1 and its cytoplasmic tail in mediating DENV infection was assessed [35,60]. TIM1 knockout cells like A549 and Huh7.5 showed significantly less DENV2 infection [60] but TIM1 mutant without a cytoplasmic tail (TIM-1 Δcyt) had no such effect when transfected in 293T cells [35]. Hence TIM1 is important for enhancing DENV infection but its cytoplasmic tail has no or minimal role in enhancing DENV infection. Nevertheless, it plays a vital role in mediating DENV internalization by clathrin-mediated endocytosis [60].

The TAM protein family is a group of three receptor protein tyrosine kinases that recognizes PS expressed on the surface of apoptotic cells indirectly via TAM ligands (Gas6 and ProS) and are expressed on phagocytes particularly DC and macrophages [35,55,57,61]. TYRO3 and AXL are two such TAM receptors that are known to recognize apoptotic cells and also enhance DENV infection [35]. Furthermore, TIM1 and AXL have been observed to be expressed in DENV permissive cell lines like A549, Vero, Cos-7 and Huh7 5.1 cells but are absent in cells which are non-permissive to DENV like 293T, U937 or RAJI cells [35]. Hence both the receptors (TIM1 and AXL) may act cooperatively and complementarily to positively influence DENV binding in the cells.

Recently a phospholipid receptor CD-300a, expressed on the surface of mast cells, monocytes and monocyte-derived macrophages (MDM) has been observed to act as a receptor of DENV [55,62]. It directly interacts with PE and to a lesser extent with PS expressed on the surface of DENV and enhances its entry in these cells [55,56]. On the expression of CD300a in HEK 293T cell line and in HeLa cells, the DENV2 infection was highly enhanced suggesting its importance as an attachment receptor but does not play a major role in mediating DENV entry [55]. In the same study, it was shown that although CD300a was expressed in monocytes, mast cells and monocyte-derived macrophages, CD300a could increase DENV infection only in MDM and was ineffective in case of other two cells suggesting its cell-specific action.

Other chaperones like Protein disulfide isomerase (PDI) has also been shown to enhance the DENV binding to the cell surface THP-1 and also in endothelial cells [58,63]. PDI is an ER-resident chaperone but has been found to be localized in various other cellular regions like a nuclear envelope, cytoplasm, Golgi, secretory vesicles and plasma membrane [64]. PDI was found to be upregulated during DENV infection in THP1 cell lines and was observed to be associated with the lipid rafts for efficient interaction with DENV. Hence, PDI plays a role in mediating DENV interaction with the susceptible cells but further studies are needed to decipher its exact role in DENV tropism as the authors failed to show direct interaction of PDI with DENV E protein [58].

Although two Fcγ receptors particularly FcγRI (CD64) and FcγRII (CD32) have been shown to mediate ADE in phagocytic cells in vitro, FcγRIIA (CD32) was found to enhance DENV infection more efficiently than FcγRIA (CD64) [37,65,66]. However, cells which facilitate ADE particularly primary monocytes, express both the cell surface receptors [37]. FcγRII has two subsets that play different roles in ADE by either activating or inhibiting the process, particularly FcγRIIA which enhances ADE and FcγRIIB which abolishes ADE and both these molecules are known to be expressed in ADE supporting cells- monocytes, macrophage and moDC (mature and immature) [7,20]. Nonetheless, it has been perceived that these cells, specifically mature moDC enlarge the ratio of FcγRIIA/FcγRIIB to facilitate DENV entry during ADE [20]. ADE hypothesis has been widely studied for monocytes, macrophages, mature DC (moDC) and mast cells but there is a high possibility of B cells and endothelial cells being involved in ADE, as they express FcγR on their cell surface [42,43,67,68,69]. Monocytes play a less significant role as primary targets for DENV since they express the lesser amount of DC-SIGN compared to DCs but has a major role to play in secondary infection during ADE, due to high levels of FcγR expression [7,10,37].

### 2.3. Mast Cells and Basophils 

Mast cells found in the dermis of the skin are important for the surveillance of the immune system and on encountering DENV it gets degranulated, secreting various cytokines (IL-1, IL-6, TNF-α and IFN-α) and chemokines (CCL5, CXCL12 and CX3CL1). This further leads to an antiviral state in nearby cells, generating an inflammatory response and cells like NK and NKT get recruited to combat DENV [70]. Various reports indicate the susceptibility of mast cells to DENV tropism and hence, mast cells are one among other cells that support DENV entry and replication [11,71]. Primary human skin mast cells were identified to support DENV2 infection as seen by qRTPCR and are also among the initial targets of DENV in the skin [71]. Furthermore, skin explants, when infected with DENV2, showed mast cells to be among the susceptible cell population showing productive infection in the dermis [11]. DENV2 was also observed in the secretory granules of these cells and were highly infectious to the uninfected cells [71]. Moreover, when these extracellular granules were injected in mice footpad, the DENV containing granules traveled through lymph to draining lymph nodes (DLN) and spleen leading to further virus dissemination. Hence, they discovered a novel mechanism used by DENV to spread in the host, starting from the skin to the various lymphoid organs, apart from being carried by the infected immune cells. 

Mast cells are FcγR bearing cells that express all the FcγRs (FcγRI, FcγRII, and FcγRIII), as observed in various cells like human cord blood-derived mast cells (CBMCs) that express all the three FcγRs, while FcγRII was prominently expressed on the surface of human mast cell lines HMC-1 and KU812 [42]. Experiments done by various groups in human mast cell-like line HMC-1 and KU812 successfully inferred the importance of heterologous antibodies to mediate DENV entry in human mast cells via FcγRII receptors [42,43,72]. These cells were infected with DENV alone or DENV with antibodies and a significant infection was observed in the presence of antibodies [42,43]. 

Apart from Fc receptors, TIM1 and TIM3 are also found to be expressed in mouse peritoneal mast cells and bone marrow-derived cultured mast cells (BMCMCs) [22,73]. These can be potential receptors used by DENV for its entry in mast cells.

### 2.4. T Cells and B Cells 

B cells and T cells have been studied by various laboratories to ascertain their roles in supporting DENV replication, but contradictory results leave it an open question to address. *In vitro* studies done on B cell lines (Raji cells, Wil 2WT, BM and LK63, Daudi and 8866) and primary B cells derived from healthy human peripheral blood mononuclear cells (PBMC) strongly state its potential role in DENV replication, both in presence and absence of heterologous antibodies [51,74,75,76]. Also, blood samples of DHF patients revealed the presence of DENV antigen in B lymphocytes [77] but the mere presence of DENV antigen does not prove DENV replication in these cells. In the humanized mouse model study, lymphocytes (both B and T cells) were DENV infected at an earlier stage of infection (1–2 dpi and 3 dpi respectively) and B cells produced important proinflammatory cytokines (IL-6 and TNF-α) similar to monocytes and macrophage [39]. Furthermore, *in vivo* experiment done on DENV infected BALB/c mice revealed the presence of NS3, E and prM in B cell follicles particularly in the germinal center (GC), B cells of DLN, indicating viral replication in these cells [67,78]. Some groups of researchers found naive primary human T cells (CD8+, CD4+) derived from PBMCs of healthy individuals and human Th and Tc clones (JK44, JK49, CB2.8 and CB6.17, HSB-2, Molt-4 and Jurkat) to be DENV permissive, producing new infectious virions from the infected cells [51,76,79,80].

In contrast to this, other groups found no evidence of DENV replication in primary B and T cells in healthy human PBMCs or in splenic B and T cells as seen by FACS analysis, RT PCR and plaque assay [37,46]. *In vivo* studies found no DENV antigen in B or T cells of infected human tissues as detected via immuno-histochemistry (IHC) and in situ hybridization (ISH) techniques [45]. Viral NS3 specific immune staining of infected AG129 mice and human tissue samples also indicated the absence of the DENV specific protein in lymphocytes of all the tissues [81]. According to Kou et al. 2008, T or B cells are not DENV permissive both in the absence or presence of facilitating anti-E antibodies. The same group showed it to be true for spleen derived mononuclear cells where splenic macrophages were infected both in the presence and absence of anti-DENV antibodies but T or B cells were uninfected [46]. Theofilopoulos et al. 1976 also reported similar results, where T cell lines lymphoblast MOLT-4 and primary T cells from healthy human PBMC were not found to support DENV replication but DENV was observed to adsorb on the cell surface early after infection. Hence, the author proposed T cells to possess the DENV receptor but due to some other intrinsic or extrinsic factors, DENV failed to enter or replicate in these cells. Schmid et al. 2014b further studied the epidermal CD45+ γδT cells in Ifnar^−/−^ mice after intradermal inoculation of DENV2 and found these cells to be non-permissive to DENV.

Contradictory reports have also been obtained regarding receptor usage of DENV for T cell entry. Heparan sulfate was identified to be the putative receptor on T cells that mediated DENV entry [79], but Bielefeldt-Ohmann et al. 2001 found no significant role of heparan sulfate in promoting DENV binding to these cells [51].

Work done on T helper (Th) cells revealed the involvement of cell surface PDI in facilitating HIV entry in these cells [82]. Studies done on PDI in relation to DENV entry have indicated it to likely play a role in DENV tropism, making it a yet another putative receptor for T cells [63] that needs further research. TIM1 and TIM 3 have been noted to be expressed primarily on the surface of Th2 cells and Th1/Tc1 cells respectively, having a role to play in both their activation and apoptosis [22,23,24,25,26,27,28,29,30,31,32,33,34,35]. Hence, these receptors might play a role in DENV entry in human T cells. No receptor has yet been identified to be responsible for DENV entry in B cells. However, FcγR might be utilized by DENV to gain access in these cells [67,69].

## 3. Discussion

Flaviviruses like Dengue virus, Japanese encephalitis virus (JEV), West Nile virus (WNV), zika virus (ZKV) and yellow fever virus (YFV) are known to show tropism with respect to diverse host cell types for propagation and thus have been shown to use multiple receptor types for undergoing cellular entry [83]. While these viruses have different pathogenic effects, yet they have partial sequence identities and similar structural features. They all are transmitted by arthropod vectors and cause diverse diseases in humans. Having single-envelope glycoprotein which are mainly responsible for virus entry, and due to many similarities amongst these different viruses, it can be hypothesized that the entry mechanisms in flaviviruses (i.e., susceptible cells and receptors) could be evolutionarily conserved, particularly with respect to at least some cell types like dendritic cells of skin and keratinocytes that are the common targets [83]. Although JEV, ZKV, DENV and YFV all infect dendritic cells, the receptors that they use have been documented as different. While JEV and ZIKA viruses use DC-SIGN, DENV and YFV, they do not use DC-SIGN to gain entry in skin dendritic cells [83]. While each of the flaviviruses might choose some cell types over the others for frequent entry and multiplication, overall, in context of flaviviruses taken together, there are many cell types that are housed by these viruses commonly with the difference lying in the type of receptors that they use to undergo cellular entry. Like JEV and WNV but unlike others, DENV has also been shown to exhibit ADE mediated infection of immune cells which pose to be a major hurdle in the field of vaccine engineering against DENV. Elaborate studies in the field of tropism and cellular entry mechanisms may thus help discover therapeutic anti-viral candidates for controlling or managing flavivirus infections.

The human immune cells that have been most extensively studied to support DENV are DC, monocytes and tissue macrophages [4,5,6,9,37,45,46,47,48]. However, recent studies have identified mast cells to be the new targets of DENV in humans and their potential in acting as viral replicating machinery [11,71]. Mast cells may be inferred to be among the early targets of DENV in the skin along with the known hosts of DENV: DCs and dermal macrophages [11,71]. Future studies will elaborate on our understanding of DENV tropism and explore other unknown targets of DENV in the host. The controversial role of lymphoid cells (T and B) in DENV tropism and replication also needs further exploration.

**Figure 1:**
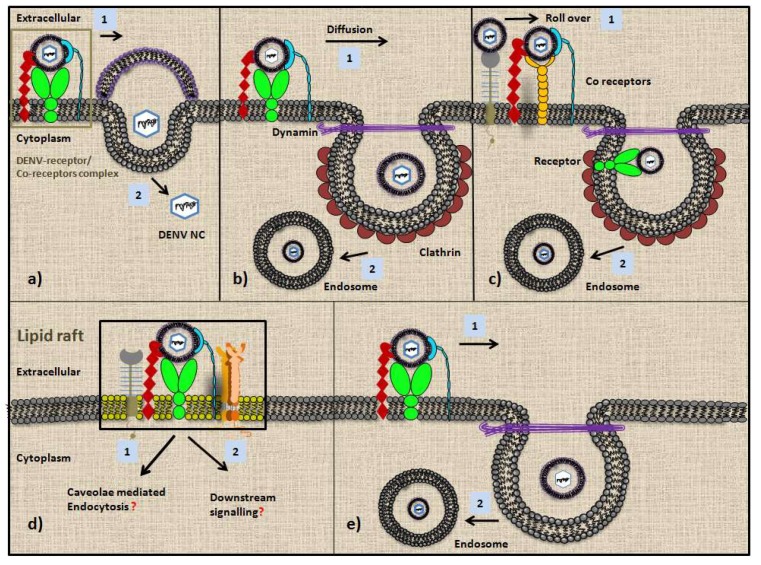
An overview of the different routes of dengue virus (DENV) entrance in susceptible cells via multiple receptors/co-receptors: (**a**) Direct entry of DENV in cells. a1) DENV first forms a complex with receptors/co-receptors and then a2) fuse with the cell membrane releasing the nucleocapsid (DENV NC) in the cytoplasm. (**b**), (**c**) Receptor-mediated endocytosis of DENV in cells in which b1) DENV after interacting with receptors and co-receptors diffuse along the membrane and enter through preformed clathrin-coated pit further leading to the b2) endosome formation or c1) DENV might possibly interact with co-receptors by rolling over the cell surface until it reaches to the main receptor present near or within the preformed clathrin-coated pit after which it gets c2) endocytosed. (**d**) Lipid rafts may also play an important role in DENV tropism by providing the platform for DENV interaction with multiple receptors and co-receptors, which in turn may lead to d1) caveolae-mediated endocytosis or d2) downstream signaling to enhance the receptor expression at the cell surface. (**e**) Non-classical, dynamin-dependent but clathrin-independent endocytosis is another route of entry that DENV might follow for ingress in cells.

Multiple receptors associated with the DENV tropism in host and vector have been studied to better understand their individual role in facilitating DENV entry. However, all studies indicate that DENV seems to utilize multiple receptors/co-receptors to gain access to the host cells and that not a single receptor can be conclusively declared to be solely involved in DENV ingress. These observations raise multiple possibilities regarding DENV-cell interaction at the entry point and the different modes of entry inside the cells (Figure 1). (a) DENV either attaches to the cell surface via its receptors/ co-receptors followed by the dissolution of the membrane (cellular and virion) at the attachment site, which further leads to the entry of the nucleocapsid in the cytoplasm directly, without the involvement of any vesicles [84,85] (Figure 1a). (b) DENV binds to the receptor/co-receptors forming DENV–receptor complex first, followed by its diffusion to the pre-formed clathrin-coated pit for endocytosis [86] (Figure 1b). (c) DENV first interacts with multiple low affinity co-receptors like DC-SIGN, langerin, CD300A, CD-14, heparan sulphate, PDI etc. via a rollover mechanism to toughen its hold on the cells, followed by its interaction with the less abundant but high affinity receptor located near the pre-existing clathrin-coated pit or within the pit after which DENV enters possibly via receptor-mediated endocytosis [86] (Figure 1c). (d) Interaction with the receptors/co-receptors in lipid rafts might mediate signal transduction to enhance the availability of the main receptor on the cell surface or might lead to caveolae-mediated endocytosis (Figure 1d). E) DENV might also follow the dynamin-dependent, clathrin-independent mode of endocytosis to enter the cells [87] (Figure 1e). There also lies a possibility that DENV deploys all these receptors in a cell-specific manner to approach the cell for a productive infection and as such does not depend on any one receptor for the entry. If this is true then it would be intriguing to know what the underlying factors behind such cell-specific interaction are. How does DENV recognize different receptors in a cell-specific way?

**Figure 2:**
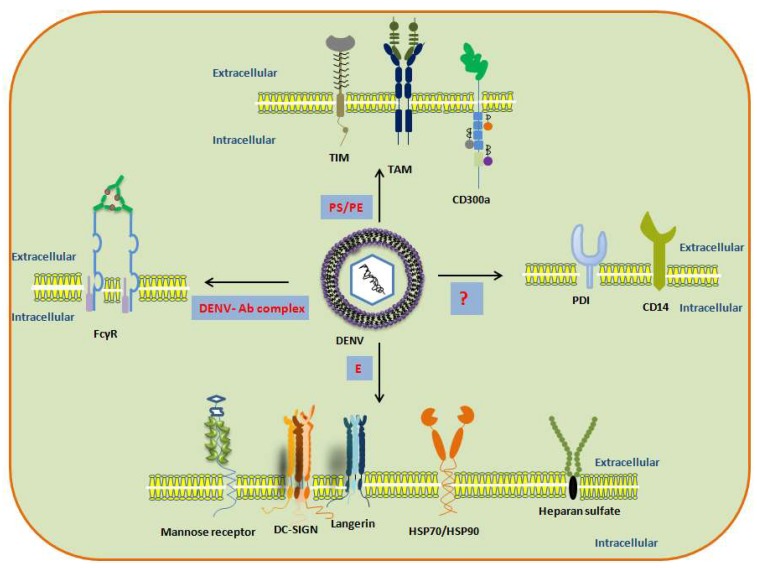
Multiple receptors used by DENV for entry in the host. PS/PE: phosphatidylserine and phosphatidylethanolamine on DENV membrane. prM: precursor membrane of DENV. E: Envelope protein of DENV. ?: Mechanism not well known.

Furthermore, as mentioned in Figure 2 and Table 1, DENV interacts with the different receptors in a cell-specific manner which further complicates the identification of a possible common receptor mandatory for the DENV entry process. MR in monocytes/macrophages and DC and recently TIM1 receptors have been shown to be important for DENV entry in these cells and these might not just be acting as co-receptors/attachment factors for DENV [8,15,17,35,60]. Further studies are needed to confirm their universal role in the ingress of DENV insusceptible cells and the detailed mechanism of the process.

Researchers have been emphasizing the importance of E protein in interacting with the host receptors, but the role of other DENV proteins has long been neglected. M protein, another important component of the DENV envelope, lies in close proximity to E protein and act as its chaperone to maintain proper E protein conformation and govern its antigenicity [88,89]. Furthermore, the glycosylation pattern of M protein has not been studied in great detail but glycosylation at Asn64–69 residues have been detected in different DENV serotypes, particularly at Asn 68 residue [16,90]. Hence, there lies a possible role of M protein in mediating DENV interaction with the host cell receptors either indirectly or directly. If it does perform any role in DENV-receptor interaction then what exactly is its role? Does it interact with any host receptor/co-receptor directly to strengthen the interaction of DENV with the target cell or it indirectly facilitates DENV E interaction with the receptors?

The true potential of DENV E in recognizing the host receptors needs to be further explored. Studies done on mosquito derived DENV E stated that apart from mannose residues at Domain II (DII) Asn67 residue, fucose, sialic acid, GalNAc and GlcNAc have also been observed at this position along with sialic acid at Domain I (DI) Asn153 site [91]. Therefore, the role of these glycan moieties in recognizing host receptors need further investigation and their binding partners may play an equally important role in attaching DENV to the cell surface. What other residues in E get glycosylated and how that affects DENV tropism in host and vector is still an open question to address.

Although structural glycoproteins are known to play a primary function in the cellular entry, the potential role of DENV non-structural (NS) proteins in modulating the entry process have also been suggested. For example, the non-structural protein NS1 has been shown to enhance DENV infection in Huh7 cells. Pre-treatment of the cells with secretory NS1 (sNS1) protein followed by DENV infection leads to an increased viral titer possibly by enhancing the endocytic ability of the cells [92]. The same group hypothesized that the increase in virus titer could be a result of triggering cell signaling pathway/s by NS1 leading to an enhanced cell surface expression of the DENV receptor. It was also hypothesized that post uptake by hepatocytes, secretory NS1 might be modulating intracellular environment so as to support viral replication and thus lead to DENV pathogenesis in liver cells. The exact cell signaling pathway/s that could influence DENV entry is yet to be explored. As compared to the envelope protein, NS1 is also highly conserved amongst the four established DENV serotypes. Thus, targeting NS1 protein could potentially help target all four serotypes. Likewise, it would be interesting to investigate the possibility of the role of other NS proteins in facilitating DENV entry in susceptible cells. Such pathways might also be targeted to design antiviral candidates to block DENV ingress in the host cells.

The focus of this review was to highlight the plethora of receptors used by DENV to infect the host which make the virus capable of infecting multiple cells in the body. We have highlighted the immune system because of the important role that it plays. The fact that DENV uses more than one receptor for its entry and hijacks diverse cell types, makes it a highly complicated system for designing antivirals or vaccine. Thus, detailed analyses of receptor usage and mediators of cellular entry for DENV might help in strategizing therapeutic interventions. Such studies might also help in developing early detection systems or improvising existing detection methodologies. Tissue-specific inhibitors could help in managing the spread of infection in Dengue patients wherein the virus could be selectively prevented gaining entry in crucial tissues like that in immune cells. Since antibody-dependent enhancement is an important limiting factor in vaccine designing for DENV, preventing virus entry in immune cells using immune cell-specific entry blockers/ anti-viral candidates could be a significant method to disrupt virus dissemination and occurrence of severe Dengue fever. Thus, combinations of tissue-specific anti-DENV antivirals along with patient care could help manage the severity of the infection and enable quicker recovery of the patients.

## Figures and Tables

**Table 1:** Outline of the various receptors that have been studied in the context of DENV entry in susceptible immune cells.

Receptors Mediating DENV Tropism	Susceptible Cells in Humans Expressing the Receptor	References
DC-SIGN	Dermal Dendritic cells (CD14+) Dermal macrophages Alveolar macrophages Dendritic cells in lungs Dendritic cells in lymph nodes Myeloid DCs in blood	Schaeffer et al. 2015 [27], Cerny et al. 2014 [9] Schaeffer et al. 2015 [27] Soilleux et al. 2003 [26] Soilleux et al. 2003 [26] Soilleux et al. 2003 [26] Sun et al. 2009 [8], Tassaneetrithep et al. 2003 [15], Wu et al. 2000 [4]
Langerin	Langerhans cells	Wu et al. 2000 [4]
Mannose receptor	Dermal Dendritic cells (CD14+, CD 1c+) Macrophages	Cerny et al. 2014 [9] Schaeffer et al. 2015 [27], Miller et al. 2008 [17]
TIM	Dendritic cells Macrophages T cells	Freeman et al. 2010 [22], Rodriguez-Manzanet et al. 2009 [23], Kobayashi et al. 2007 [24] Freeman et al. 2010 [22], Rodriguez-Manzanet et al. 2009 [23] Freeman et al. 2010 [22], Rodriguez-Manzanet et al. 2009 [23], Kobayashi et al. 2007 [24], Meertens et al. 2012 [35]
TAM	Langerhans cells Macrophages	Bauer et al. 2012 [25], Lemke et al. 2008 [57] Lemke et al. 2008 [57]
CD-300a	Mast cells Monocyte-derived macrophages Blood monocytes	Borrego et al. 2013 [62], Carnec et al. 2015 [55] Borrego et al. 2013 [62], Carnec et al. 2015 [55] Borrego et al. 2013 [62], Carnec et al. 2015 [55]
FcγR	Mast cells Macrophages Blood Monocytes Conventional DC B cells	Brown et al. 2006 [42], King et al. 2000 [43], Brown et al. 2011 [72] Boonnak et al. 2008 [20], Schmid et al. 2014b [10] Kou et al. 2008 [37], Boonnak et al. 2008 [20], Schmid et al. 2014b [10] Boonnak et al. 2008 [20], Schmid et al. 2014b [10] Gergely et al. 1977 [69]
Protin Disulfide Isomerase (PDI)	Blood Monocytes T cells	Diwaker et al. 2015 [58] Barbouche et al. 2005 [82]
Heat shock proteins (HSP70,HSP 90)	Blood Monocytes	Reyes-del Valle et al. 2005 [54]
Heparan Sulfate	T cells	Silveira et al. 2018 [79], Bielefeldt-Ohmann et al. 2001 [51]
CD-14	Macrophages Blood Monocytes	Wright et al. 1990 [59] Wright et al. 1990 [59]

## References

[1] Souza H.F., Da Silva Almeida B., Boscardin S.B. (2016). Early dengue virus interactions: The role of dendritic cells during infection. Virus Res..

[2] Mukhopadhyay S., Kuhn R.J., Rossmann M.G. (2005). A structural perspective of the flavivirus life cycle. Nat. Rev. Microbiol..

[3] Martin B.E., Koraka P., Osterhaus A.D. (2009). Dengue virus pathogenesis: An integrated view. Clin. Microbiol. Rev..

[4] Wu S.J., Grouard-Vogel G., Sun W., Mascola J.R., Brachtel E., Putvatana R., Louder M.K., Filgueira L., Marovich M.A., Wong H.K. (2000). Human skin Langerhans cells are targets of dengue virus infection. Nat. Med..

[5] Marovich M., Grouard-Vogel G., Eller M., Tassaneetrithep B., Birx D., Hayes C., Schlesinger-Frankel S., Louder M., Mascola J., Sun W. (2001). Human dendritic cells as targets of dengue virus infection. J. Investig. Derm. Symp. Proc..

[6] Ho L.J., Wang J.J., Shaio M.F., Kao C.L., Chang D.M., Han S.W., Lai J.H. (2001). Infection of human dendritic cells by dengue virus causes cell maturation and cytokine production. J. Immunol..

[7] Schmid M.A., Diamond M.S., Harris E. (2014). Dendritic cells in dengue virus infection: Targets of virus replication and mediators of immunity. Front. Immunol..

[8] Sun P., Fernandez S., Marovich M.A., Palmer D.R., Celluzzi C.M., Boonnak K., Liang Z., Subramanian H., Porter K.R., Sun W. (2009). Functional characterization of *ex vivo* blood myeloid and plasmacytoid dendritic cells after infection with dengue virus. Virology.

[9] Cerny D., Haniffa M., Shin A., Bigliardi P., Tan B.K., Lee B., Poidinger M., Tan E.Y., Ginhoux F., Fink K. (2014). Selective susceptibility of human skin antigen presenting cells to productive dengue virus infection. PLoS Pathog..

[10] Schmid M.A., Harris E. (2014). Monocyte recruitment to the dermis and differentiation to dendritic cells increases the targets for dengue virus replication. PLoS Pathog..

[11] Duangkhae P., Erdos G., Ryman K.D., Watkins S.C., Falo L.D., Marques E.T., Barratt-Boyes S.M. (2018). Interplay between keratinocytes and myeloid cells drives dengue virus spread in human skin. J. Investig. Derm..

[12] Merad M., Manz M.G., Karsunky H., Wagers A., Peters W., Charo I., Weissman I.L., Cyster J.G., Engleman E.G. (2002). Langerhans cells renew in the skin throughout life under steady-state conditions. Nat. Immunol..

[13] Liu P., Ridilla M., Patel P., Betts L., Gallichotte E., Shahidi L., Thompson N.L., Jacobson K. (2017). Beyond attachment: Roles of DC-SIGN in dengue virus infection. Traffic.

[14] Sun P., Kochel T.J. (2013). The battle between infection and host immune responses of dengue virus and its implication in dengue disease pathogenesis. Sci. World J..

[15] Tassaneetrithep B., Burgess T.H., Granelli-Piperno A., Trumpfheller C., Finke J., Sun W., Eller M.A., Pattanapanyasat K., Sarasombath S., Birx D.L. (2003). DC-SIGN (CD209) mediates dengue virus infection of human dendritic cells. J. Exp. Med..

[16] Yap S.S., Nguyen-Khuong T., Rudd P.M., Alonso S. (2017). Dengue virus glycosylation: What do we know?. Front. Microbiol..

[17] Miller J.L., M deWet B.J., Martinez-Pomares L., Radcliffe C.M., Dwek R.A., Rudd P.M., Gordon S. (2008). The mannose receptor mediates dengue virus infection of macrophages. PLoS Pathog..

[18] Valladeau J., Duvert-Frances V., Pin J.J., Dezutter-Dambuyant C., Vincent C., Massacrier C., Vincent J., Yoneda K., Banchereau J., Caux C. (1999). The monoclonal antibody DCGM4 recognizes Langerin, a protein specific of Langerhans cells, and is rapidly internalized from the cell surface. Eur. J. Immunol..

[19] Ingber A. (2007). Langerhans cell receptors. Derm. Clin..

[20] Boonnak K., Slike B.M., Burgess T.H., Mason R.M., Wu S.J., Sun P., Porter K., Rudiman I.F., Yuwono D., Puthavathana P. (2008). Role of dendritic cells in antibody-dependent enhancement of dengue virus infection. J. Virol..

[21] Boonnak K., Dambach K.M., Donofriom G.C., Tassaneetrithep B., Marovich M.A. (2011). Cell type specificity and host genetic polymorphisms influence antibody-dependent enhancement of dengue virus infection. J. Virol..

[22] Freeman G.J., Casasnovas J.M., Umetsu D.T., DeKruyff R.H. (2010). TIM genes: A family of cell surface phosphatidylserine receptors that regulate innate and adaptive immunity. Immunol. Rev..

[23] Rodriguez-Manzanet R., DeKruyff R., Kuchroo V.K., Umetsu D.T. (2009). The costimulatory role of TIM molecules. Immunol. Rev..

[24] Kobayashi N., Karisola P., Peña-Cruz V., Dorfman D.M., Jinushi M., Umetsu S.E., Butte M.J., Nagumo H., Chernova I., Zhu B. (2007). TIM-1 and TIM-4 glycoproteins bind phosphatidylserine and mediate uptake of apoptotic cells. Immunity.

[25] Bauer T., Zagórska A., Jurkin J., Yasmin N., Köffel R., Richter S., Gesslbauer B., Lemke G., Strobl H. (2012). Identification of Axl as a downstream effector of TGF-β1 during Langerhans cell differentiation and epidermal homeostasis. J. Exp. Med..

[26] Soilleux E.J. (2003). DC-SIGN (dendritic cell-specific ICAM-grabbing non-integrin) and DC-SIGN-related (DC-SIGNR): Friend or foe?. Clin. Sci..

[27] Schaeffer E., Flacher V., Papageorgiou V., Decossas M., Fauny J.D., Krämer M., Mueller C.G. (2015). Dermal CD14+ dendritic cell and macrophage infection by dengue virus is stimulated by interleukin-4. J. Investig. Dermatol..

[28] Lozach P.Y., Burleigh L., Staropoli I., Navarro-Sanchez E., Harriague J., Virelizier J.L., Rey F.A., Desprès P., Arenzana-Seisdedos F., Amara A. (2005). Dendritic cell-specific intercellular adhesion molecule 3-grabbing non-integrin (DC-SIGN)-mediated enhancement of dengue virus infection is independent of DC-SIGN internalization signals. J. Biol. Chem..

[29] Pokidysheva E., Zhang Y., Battisti A.J., Bator-Kelly C.M., Chipman P.R., Xiao C., Gregorio G.G., Hendrickson W.A., Kuhn R.J., Rossmann M.G. (2006). Cryo-EM reconstruction of dengue virus in complex with the carbohydrate recognition domain of DC-SIGN. Cell.

[30] Dubayle J., Vialle S., Schneider D., Pontvianne J., Mantel N., Adam O., Guy B., Talaga P. (2015). Site-specific characterization of envelope protein N-glycosylation on Sanofi Pasteur’s tetravalent CYD dengue vaccine. Vaccine.

[31] Smit J., Moesker B., Rodenhuis-Zybert I., Wilschut J. (2011). Flavivirus cell entry and membrane fusion. Viruses.

[32] Guo Y., Feinberg H., Conroy E., Mitchell D.A., Alvarez R., Blixt O., Taylor M.E., Weis W.I., Drickamer K. (2004). Structural basis for distinct ligand-binding and targeting properties of the receptors DC-SIGN and DC-SIGNR. Nat. Struct. Mol. Biol..

[33] Acosta E.G., Talarico L.B., Damonte E.B. (2008). Cell entry of dengue virus. Future Virol..

[34] Geijtenbeek T.B., Gringhuis S.I. (2009). Signalling through C-type lectin receptors: Shaping immune responses. Nat. Rev. Immunol..

[35] Meertens L., Carnec X., Lecoin M.P., Ramdasi R., Guivel-Benhassine F., Lew E., Lemke G., Schwartz O., Amara A. (2012). The TIM and TAM families of phosphatidylserine receptors mediate dengue virus entry. Cell Host Microbe..

[36] Kurane I. (2007). Dengue hemorrhagic fever with special emphasis on immunopathogenesis. Comp. Immunol. Microbiol. Infect. Dis..

[37] Kou Z., Quinn M., Chen H., Rodrigo W.S., Rose R.C., Schlesinger J.J., Jin X. (2008). Monocytes, but not T or B cells, are the principal target cells for dengue virus (DV) infection among human peripheral blood mononuclear cells. J. Med. Virol..

[38] Simmons C.P., Farrar J.J., van Vinh Chau N., Wills B. (2012). Dengue. New Engl. J. Med..

[39] Mota J., Rico-Hesse R. (2011). Dengue virus tropism in humanized mice recapitulates human dengue fever. PLoS ONE.

[40] Priyamvada L., Cho A., Onlamoon N., Zheng N.Y., Huang M., Kovalenkov Y., Chokephaibulkit K., Angkasekwinai N., Pattanapanyasat K., Ahmed R. (2016). B cell responses during secondary dengue virus infection are dominated by highly cross-reactive, memory-derived plasmablasts. J. Virol..

[41] Halstead S.B., Mahalingam S., Marovich M.A., Ubol S., Mosser DM. (2010). Intrinsic antibody-dependent enhancement of microbial infection in macrophages: Disease regulation by immune complexes. Lancet Infect. Dis..

[42] Brown M.G., King C.A., Sherren C., Marshall J.S., Anderson R. (2006). A dominant role for FcγRII in antibody-enhanced dengue virus infection of human mast cells and associated CCL5 release. J. Leukoc. Biol..

[43] King C.A., Marshall J.S., Alshurafa H., Anderson R. (2000). Release of vasoactive cytokines by antibody-enhanced dengue virus infection of a human mast cell/basophil line. J. Virol..

[44] Flipse J., Torres S., Diosa-Toro M., van der Ende-Metselaar H., Herrera-Rodriguez J., Urcuqui-Inchima S., Huckriede A., Rodenhuis-Zybert I.A., Smit J.M. (2016). Dengue tropism for macrophages and dendritic cells: The host cell effect. J. Gen. Virol..

[45] Jessie K., Fong M.Y., Devi S., Lam S.K., Wong K.T. (2004). Localization of dengue virus in naturally infected human tissues, by immunohistochemistry and in situ hybridization. J. Infect. Dis..

[46] Blackley S., Kou Z., Chen H., Quinn M., Rose R.C., Schlesinger J.J., Coppage M., Jin X. (2007). Primary human splenic macrophages, but not T or B cells, are the principal target cells for dengue virus infection in vitro. J. Virol..

[47] Prestwood T.R., May M.M., Plummer E.M., Morar M.M., Yauch L.E., Shresta S. (2012). Trafficking and replication patterns reveal splenic macrophages as major targets of dengue virus in mice. J. Virol..

[48] Jhan M.K., Tsai T.T., Chen C.L., Tsai C.C., Cheng Y.L., Lee Y.C., Ko C.Y., Lin Y.S., Chang C.P., Lin L.T. (2017). Dengue virus infection increases microglial cell migration. Sci. Rep..

[49] Ginhoux F., Tacke F., Angeli V., Bogunovic M., Loubeau M., Dai X.M., Stanley E.R., Randolph G.J., Merad M. (2006). Langerhans cells arise from monocytes *in vivo*. Nat. Immunol..

[50] Lo Y.L., Liou G.G., Lyu J.H., Hsiao M., Hsu T.L., Wong C.H. (2016). Dengue virus infection is through a cooperative interaction between a mannose receptor and CLEC5A on macrophage as a multivalent hetero-complex. PLoS ONE.

[51] Bielefeldt-Ohmann H., Meyer M., Fitzpatrick D.R., Mackenzie J.S. (2001). Dengue virus binding to human leukocyte cell lines: Receptor usage differs between cell types and virus strains. Virus Res..

[52] Chen Y.C., Wang S.Y., King C.C. (1999). Bacterial lipopolysaccharide inhibits dengue virus infection of primary human monocytes/macrophages by blockade of virus entry via a CD14-dependent mechanism. J. Virol..

[53] Reyes-del Valle J., Salas-Benito J., Soto-Acosta R., del Angel R.M. (2014). Dengue virus cellular receptors and tropism. Curr. Trop. Med. Rep..

[54] Reyes-del Valle J., Chávez-Salinas S., Medina F., Del Angel R.M. (2005). Heat shock protein 90 and heat shock protein 70 are components of dengue virus receptor complex in human cells. J. Virol..

[55] Carnec X., Meertens L., Dejarnac O., Perera-Lecoin M., Hafirassou M.L., Kitaura J., Ramdasi R., Schwartz O., Amara A. (2015). The phosphatidylserine and phosphatidylethanolamine receptor CD300a binds dengue virus and enhances infection. J. Virol..

[56] Simhadri V.R., Andersen J.F., Calvo E., Choi S.C., Coligan J.E., Borrego F. (2012). Human CD300a binds to phosphatidylethanolamine and phosphatidylserine, and modulates the phagocytosis of dead cells. Blood.

[57] Lemke G., Rothlin C.V. (2008). Immunobiology of the TAM receptors. Nat. Rev. Immunol..

[58] Diwaker D., Mishra K.P., Ganju L., Singh S.B. (2015). Protein disulfide isomerase mediates dengue virus entry in association with lipid rafts. Viral Immunol..

[59] Wright S.D., Ramos R.A., Tobias P.S., Ulevitch R.J., Mathison J.C. (1990). CD14, a receptor for complexes of lipopolysaccharide (LPS) and LPS binding protein. Science.

[60] Dejarnac O., Hafirassou M.L., Chazal M., Versapuech M., Gaillard J., Perera-Lecoin M., Umana-Diaz C., Bonnet-Madin L., Carnec X., Tinevez J.Y. (2018). TIM-1 ubiquitination mediates dengue virus entry. Cell Rep..

[61] Fernández-Fernández L., Bellido-Martín L., de Frutos P.G. (2008). Growth arrest-specific gene 6 (GAS6). Thromb. Haemost..

[62] Borrego F. (2013). The CD300 molecules: An emerging family of regulators of the immune system. Blood.

[63] Wan S.W., Lin C.F., Lu Y.T., Lei H.Y., Anderson R., Lin Y.S. (2012). Endothelial cell surface expression of protein disulfide isomerase activates β1 and β3 integrins and facilitates dengue virus infection. J. Cell. Biochem..

[64] Turano C., Coppari S., Altieri F., Ferraro A. (2002). Proteins of the PDI family: Unpredicted non-ER locations and functions. J. Cell. Physiol..

[65] Rodrigo W.S., Jin X., Blackley S.D., Rose R.C., Schlesinger J.J. (2006). Differential enhancement of dengue virus immune complex infectivity mediated by signaling-competent and signaling-incompetent human FcγRIA (CD64) or FcγRIIA (CD32). J. Virol..

[66] Puerta-Guardo H., Mosso C., Medina F., Liprandi F., Ludert J.E., del Angel R.M. (2010). Antibody-dependent enhancement of dengue virus infection in U937 cells requires cholesterol-rich membrane microdomains. J. Gen. Virol..

[67] Yam-Puc J.C., Cedillo-Barrón L., Aguilar-Medina E.M., Ramos-Payan R., Escobar-Gutiérrez A., Flores-Romo L. (2016). The cellular bases of antibody responses during dengue virus infection. Front. Immunol..

[68] Zellweger R.L.M., Prestwood T.R., Shresta S. (2010). Enhanced infection of liver sinusoidal endothelial cells in a mouse model Of antibody-induced severe dengue disease. Cell Host Microbe..

[69] Gergely P., Bakács T., Cornain S., Klein E. (1977). Fc receptors on human blood B lymphocytes. Clin. Exp. Immunol..

[70] John A.L., Rathore A.P., Yap H., Ng M.L., Metcalfe D.D., Vasudevan S.G., Abraham S.N. (2011). Immune surveillance by mast cells during dengue infection promotes natural killer (NK) and NKT-cell recruitment and viral clearance. Proc. Natl. Acad. Sci. USA.

[71] Troupin A., Shirley D., Londono-Renteria B., Watson A.M., McHale C., Hall A., Hartstone-Rose A., Klimstra W.B., Gomez G., Colpitts T.M. (2016). A role for human skin mast cells in dengue virus infection and systemic spread. J. Immunol..

[72] Brown M.G., Hermann L.L., Issekutz A.C., Marshall J.S., Rowter D., Al-Afif A., Anderson R. (2011). Dengue virus infection of mast cells triggers endothelial cell activation. J. Virol..

[73] Nakae S., Iikura M., Suto H., Akiba H., Umetsu D.T., DeKruyff R.H., Saito H., Galli S.J. (2007). TIM-1 and TIM-3 enhancement of Th2 cytokine production by mast cells. Blood.

[74] Lin Y.W., Wang K.J., Lei H.Y., Lin Y.S., Yeh T.M., Liu H.S., Liu C.C., Chen S.H. (2002). Virus replication and cytokine production in dengue virus-infected human B lymphocytes. J. Virol..

[75] Theofilopoulos A.N., Brandt W.E., Russell P.K., Dixon F.T. (1976). Replication of dengue-2 virus in cultured human lymphoblastoid cells and subpopulations of human peripheral leukocytes. J. Immunol..

[76] Kurane I., Kontny U., Janus J., Ennis F.A. (1990). Dengue-2 virus infection of human mononuclear cell lines and establishment of persistent infections. Arch. Virol..

[77] Boonpucknavig S., Bhamarapravati N., Nimmannitya S., Phalavadhtana A., Siripont J. (1976). Immunofluorescent staining of the surfaces of lymphocytes in suspension from patients with dengue hemorrhagic fever. Am. J. Pathol..

[78] Yam-Puc J.C., García-Cordero J., Calderón-Amador J., Donis-Maturano L., Cedillo-Barrón L., Flores-Romo L. (2015). Germinal center reaction following cutaneous dengue virus infection in immune-competent mice. Front. Immunol..

[79] Silveira G.F., Wowk P.F., Cataneo A.H., dos Santos P.F., Delgobo M., Stimamiglio M.A., Sarzi M.L., Thomazelli A.P., Conchon-Costa I., Pavanelli W.R. (2018). Human T lymphocytes are permissive for dengue virus replication. J. Virol..

[80] Mentor N.A., Kurane I. (1997). Dengue virus infection of human T lymphocytes. Acta Virol..

[81] Balsitis S.J., Coloma J., Castro G., Alava A., Flores D., McKerrow J.H., Beatty P.R., Harris E. (2009). Tropism of dengue virus in mice and humans defined by viral nonstructural protein 3-specific immunostaining. Am. J. Trop. Med. Hyg..

[82] Barbouche R., Lortat-Jacob H., Jones I.M., Fenouillet E. (2005). Glycosaminoglycans and protein disulfide isomerase-mediated reduction of HIV Env. Mol. Pharmacol..

[83] Laureti M., Narayanan D., Rodriguez-Andres J., Fazakerley J.K., Kedzierski L. (2018). *Flavivirus* Receptors: Diversity, Identity, and Cell Entry. Front. Immunol..

[84] Hase T., Summers P.L., Eckels K.H. (1989). *Flavivirus* entry into cultured mosquito cells and human peripheral blood monocytes. Arch. Virol..

[85] Lim H.Y., Ng M.L. (1999). A different mode of entry by dengue-2 neutralisation escape mutant virus. Arch. Virol..

[86] Van der Schaar H.M., Rust M.J., Chen C., van der Ende-Metselaar H., Wilschut J., Zhuang X., Smit J.M. (2008). Dissecting the cell entry pathway of dengue virus by single-particle tracking in living cells. PLoS Pathog..

[87] Acosta E.G., Castilla V., Damonte E.B. (2009). Alternative infectious entry pathways for dengue virus serotypes into mammalian cells. Cell. Microbiol..

[88] Heinz F.X., Stiasny K., Püschner-Auer G., Holzmann H., Allison S.L., Mandl C.W., Kunz C. (1994). Structural changes and functional control of the tick-borne encephalitis virus glycoprotein E by the heterodimeric association with protein prM. Virology.

[89] Aruna R. (2014). Review on dengue viral replication, assembly and entry into the host cells. Int. J. Curr. Microbiol. Appl. Sci..

[90] Courageot M.P., Frenkiel M.P., Dos Santos C.D., Deubel V., Desprès P. (2000). α-Glucosidase inhibitors reduce dengue virus production by affecting the initial steps of virion morphogenesis in the endoplasmic reticulum. J. Virol..

[91] Lei Y., Yu H., Dong Y., Yang J., Ye W., Wang Y., Chen W., Jia Z., Xu Z., Li Z. (2015). Characterization of N-glycan structures on the surface of mature dengue 2 virus derived from insect cells. PLoS ONE.

[92] Alcon-LePoder S., Drouet M.T., Roux P., Frenkiel M.P., Arborio M., Durand-Schneider A.M., Maurice M., Le Blanc I., Gruenberg J., Flamand M. (2005). The secreted form of dengue virus non-structural protein NS1 is endocytosed by hepatocytes and accumulates in late endosomes: Implications for viral infectivity. J. Virol..

